# Don’t Forget the Humble Text Message: 25 Years of Text Messaging in Health

**DOI:** 10.2196/59888

**Published:** 2024-12-17

**Authors:** Rosie Dobson, Robyn Whittaker, Lorien C Abroms, Dale Bramley, Caroline Free, Hayden McRobbie, Melanie Stowell, Anthony Rodgers

**Affiliations:** 1 School of Population Health University of Auckland Auckland New Zealand; 2 Service Improvement and Innovation Te Whatu Ora Auckland New Zealand; 3 Department of Prevention and Community Health Milken Institute School of Public Health The George Washington University Washington, DC, DC United States; 4 Clinical Trials Unit London School of Hygiene & Tropical Medicine London United Kingdom; 5 National Public Health Service Te Whatu Ora Auckland New Zealand; 6 Faculty of Medicine & Health University of New South Wales Sydney Australia; 7 The George Institute for Global Health Sydney Australia

**Keywords:** text messaging, messaging, SMS, texting, mHealth, mobile health

## Abstract

Since the early studies exploring the use of SMS text messaging for health intervention, text messaging has played a pivotal role in the advancement of mobile health. As an intervention modality, text messaging has provided vital learnings for the design and delivery of interventions, particularly in low-resource settings. Despite the advances in technology over the last 25 years, text messaging is still being used in largely the same way to deliver health information, behavior change interventions, and support. The strong, consistent evidence for the benefits of this type of intervention has made text messaging a routine part of health interventions around the world. Key to its success is its simplicity, alongside the benefit of being arguably the most accessible form of consumer digital health intervention. Text message interventions are well suited for public health interventions due to their low cost, vast reach, frequent use, high read rates, and ability to be tailored and personalized. Furthermore, the nature of text messaging interventions makes them ideal for the delivery of multilingual, culturally tailored interventions, which is important in the context of increasing cultural diversity in many countries internationally. Indeed, studies assessing text message–based health interventions have shown them to be effective across sociodemographic and ethnic groups and have led to their adoption into national-level health promotion programs. With a growing focus on artificial intelligence, robotics, sensors, and other advances in digital health, there is an opportunity to integrate these technologies into text messaging programs. Simultaneously, it is essential that equity remains at the forefront for digital health researchers, developers, and implementers. Ensuring digital health solutions address inequities in health experienced across the world while taking action to maximize digital inclusion will ensure the true potential of digital health is realized. Text messaging has the potential to continue to play a pivotal role in the delivery of equitable digital health tools to communities around the world for many years to come. Further new technologies can build on the humble text message, leveraging its success to advance the field of digital health. This Viewpoint presents a retrospective of text messaging in health, drawing on the example of text message–based interventions for smoking cessation, and presents evidence for the continued relevance of this mobile health modality in 2025 and beyond.

## Introduction

In December 1992, the first SMS text message was sent. It contained the words “Merry Christmas” and was sent by a 22-year-old software programmer from the United Kingdom [1]. It took several more years for text messaging to become a standard means of communication from mobile phone to mobile phone. Now, as the most widely used feature on mobile phones, more than 5 billion people send and receive text messages globally [1]. With the rise in messaging apps such as WhatsApp and WeChat, the end of the text message was envisioned, but contrary to expectations, text messages have still remained a key form of communication, with around 40 billion messages sent every day around the world [1]. Their ubiquity is not restricted to high-income contexts, with mobile phone penetration high internationally across demographic groups [2]. People tend to not ignore text messages, with 95% of all text messages opened and read within 3 minutes of being received [3], enabling text messages to be effective prompts. In comparison, email open rates average just 20% [4]. Text messages also have the greatest response rate—209% higher than phone calls, email, or social media apps like Facebook [3]. Coupled with their low cost to implement, this makes the humble text message a valuable tool for health services [5].

Text messaging has been used to support or deliver health services and programs for approximately 25 years [5]. Early research agendas included testing the use of text messaging to communicate health information, such as coordination of health professionals’ schedules, patient appointment reminders, delivery of patient test results, and monitoring of posttreatment side effects [6]. The first published study using text messaging for health promotion is said to be from 2002 [5,7]; it consisted of a program to support young people in managing their asthma with daily reminders to use an inhaler, health education tips, and safety messages [8]. Publications on the topic increased in the following years, with the first randomized controlled trial (RCT) published in 2005 [9], and the first systematic review on text messaging interventions for health published in 2009 [6,7]. This review assessed findings from 14 studies, most of which focused on text messaging to support clinical care (eg, diabetes self-management [6]).

This Viewpoint presents a reflection on text messaging in health and discusses why this modality is still relevant today despite the rapid advances in technology in health. We will use an example of a text message–based intervention for smoking cessation (mCessation), one of the first areas to use text messaging as a stand-alone health program with good success—and one that our team have been involved in for 25 years [9].

## The Example of mCessation

Our foray into this work began when Prof Anthony Rodgers noticed that people instinctively reached for either their cigarettes or their mobile phones after a meal, and the idea of replacing one with the other to help people quit smoking was born. Professor Rodgers started piecing together funding (including funding from the National Heart Foundation of New Zealand, the Cancer Society of New Zealand, Vodafone New Zealand, and Alcatel) and a team who used the then-standard “brief intervention” training to turn what cessation counselors would say into short, snappy text messages (with a 160-character maximum for SMS text messages at the time). This eventually led to the first published RCT of a health intervention, Stop Smoking by Mobile Phone (STOMP), delivered solely by text messages in 2005 [9]. This national RCT of 1100 people across New Zealand demonstrated a doubling of quit rates compared with no support regardless of age, socioeconomic status, geographic location, or level of addiction. It was important to show that STOMP was as effective for Māori—the Indigenous population of New Zealand—as non-Māori due to the high smoking rates and health consequences in this population, as well as the obligations of the health service to uphold Te Tiriti o Waitangi (the Treaty of Waitangi) [10]. Indeed, the intervention was shown to be as effective for Māori as non-Māori at increasing short-term (6 weeks) self-reported quit rates.

This study represents the start of a large program of work in mCessation that continues today. The first steps were working with the New Zealand Ministry of Health and the Quit Group, a New Zealand service delivering smoking cessation programs, to implement the intervention as a national smoking cessation program. Real-world evaluations of this national service found it achieved quit rates comparable to the National Quitline but was less expensive to deliver [11]. At the same time there was an international collaboration with the London School of Hygiene & Tropical Medicine (LSHTM) to adapt the messages for a UK population, pilot test [12], and then conduct a full RCT across England (funded by the UK Medical Research Council, Cancer Research UK, and Primary Care Research Networks). The text-messaging intervention, txt2stop, was delivered from Auckland, New Zealand, to 2915 intervention group participants recruited by the LSHTM [13]. This high-quality RCT won the Royal College of General Practitioners paper of the year award in 2011 [14]. The trial repeated New Zealand’s findings of doubling quit rates and included biochemical verification of quitting and a health economic analysis [15] that showed it to be one of the most cost-effective health interventions and potentially cost-saving for the National Health Service.

From there, the number of studies increased, and we conducted the first (and following) Cochrane systematic reviews of the use of mobile phones for smoking cessation [16-19]. This confirmed the short-term effectiveness of text message program trials in self-reported quitting. The most recent revision of the Cochrane review, completed in 2019, found moderate-certainty evidence that text message–based smoking cessation interventions result in greater quit rates at 6-12 months than minimal smoking cessation support, as well as interventions that use text message–based interventions in addition to other forms of support, compared to other forms of support alone [19]. Studies have also documented promising results for populations at higher risk for adverse health outcomes, such as pregnant smokers [20] and smokers who are HIV positive [21], as well as for smokers in low- and middle-income countries [22-24].

In 2015 the World Health Organization (WHO) picked up mCessation as the first program in its “Be He@lthy, Be Mobile” global initiative with the International Telecommunication Union, citing the demonstrated effectiveness of studies like STOMP and txt2stop as a rationale for scaling up mobile phone–based support for smoking cessation [25]. The handbook provides a detailed work plan and set of messages from the US National Cancer Institute. The WHO is working directly with several countries (eg, Burkina Faso, China, and the Philippines) to help them implement large-scale smoking cessation programs [26]. One example is India, which implemented a nationwide mCessation program in 2015 and saw a self-reported quit rate of 19% and a quit attempt rate of 66% among registered subscribers [27].

There have been many more studies collated in updates of the Cochrane reviews. These have tended to show the same results over time and support further adoption of large-scale mCessation programs, such as those offered by the US National Cancer Institute [28,29]. Moreover, despite newer technologies in this space, text messaging–based smoking cessation has not been surpassed by other forms of digital health, such as app-based interventions—though more research is needed to adequately compare the relative effectiveness of digital modalities for smoking cessation [19,30].

But the success of text messaging hasn’t just been limited to smoking cessation. Other areas of health have caught on, with success shown in studies assessing text messaging to support medication and appointment adherence [31,32], maternal health [33], diabetes management [34], sexual health [35,36], cancer screening [37] and blood pressure control [38], to name a few.

## So Why Is the Simple Text Message So Useful for the Delivery of Health Interventions?

The reasons for this are likely multifaceted but may include text messaging’s reach into nearly all population groups, its timeliness, its seamless integration into daily habits (eg, checking one’s mobile phone), its alignment with key behavior change techniques, and its ease of adaptation and personalization.

Text messaging remains the most equitable way of reaching people regardless of type of phone, socioeconomic status, or geographic location. In much of the world, it is free to receive text messages and a smartphone is not required to receive a text message. Further, recipient replies to text messages can be “zero-rated” by health program providers so that they don’t cost the individual, ensuring access across socioeconomic groups. Even so, evidence shows that text message campaigns are a cost-effective way to reach people [15,39].

Mobile phones have become integral to everyday life for the majority of people on the planet. Furthermore, people are remarkably well trained to look at their text messages within minutes of receiving them, and unlike notifications from apps or platforms, they do not require high levels of digital literacy and are relatively difficult to disregard. With a device so integral to daily life, using this modality for health delivery and health promotion and behavior change interventions makes sense.

Text messages align well with behavior change techniques that have been tried and tested over time [40]. Unprompted, brief text messages are useful as reminders of the user’s behavior change goal, providing positive reinforcement of engagement with health behaviors, delivering health information in simple and digestible bite-sized amounts, and providing motivation and support without requiring the end user to actively engage or seek further information or support to maintain engagement [41]. It is well established that personalized health information alone is not sufficient to change behavior [42], and therefore the success of text messaging likely lies in the interplay between the content and the delivery mechanism—that is, just the fact that messages can be scheduled to arrive regularly, at key times or randomly, and in one word or glance they can be associated with the behavior in question. Indeed, prior research has shown that merely their ability to remind people of the targeted behavior change goal is highly valued by users [43]. Beyond the content and delivery mechanism, other intervention characteristics (eg, duration and dosage) may also be key factors in the success of text messaging interventions. Further, there is growing evidence for the positive impact of participatory design methods in the development of text messaging interventions (eg, Dobson et al [44]) that allow for intervention characteristics to be individually tailored.

The nature of text message–based interventions makes them ideal for the delivery of culturally tailored and personalized interventions, which has been identified as a key element of effective technology-based smoking cessation programs [30,45]. They can be adapted for different populations and contexts, translated into multiple languages, and personalized with peoples’ names and key information. An example of this is in maternal health, where a text message–based program developed by our team delivers free, culturally tailored, and personalized information and support to pregnant individuals and their families. Participants can receive texts during pregnancy through the first 2 years of a child’s life [46]. The program was originally designed to increase reach to populations in the Auckland region that were being missed by traditional New Zealand maternal support. There are 16 different versions of the Text for MATernal and Child Health (TextMATCH) program, accommodating different languages, cultures, and relationships to the child (mother or family member); this has resulted in a library of over 6000 messages. The program is tailored to the recipient’s culture, language preference, and stage of pregnancy or baby’s age, and it is personalized to the individual by using information such as the person’s name and their baby’s name. Since being implemented 10 years ago, over 8000 women and family members have received the program, and it has been adapted for other regions in New Zealand [47].

Many of the advantages of text messaging are also mirrored in app-based messaging, such as on WeChat, WhatsApp, and Facebook Messenger. Although less consistently used across demographic groups, many of these tools are highly used in targeted populations (eg, WeChat in China), making them arguably more useful for health interventions in these populations than traditional text messaging [48]. But there are a number of differences between app-based messaging and text messaging that raise concerns related to equity and reach, such as the need for data or Wi-Fi, device capacity requirements, data privacy, and, in many cases, the requirement for a subscription to an additional product [49]. Furthermore, text messages are less likely to be muted than app-based messages [50], as people increasingly experience “notification fatigue” from apps [51].

Although the evidence for text messaging appears strong, particularly in smoking cessation, it is important to note that not all studies of text messaging interventions have had consistent positive results (eg, Redfern et al [52]). There is a need for research to understand the mechanisms for success with this modality, including the degree to which the different aspects of the design, including the content, tailoring, and personalization, impact effectiveness and engagement, as well as understanding the most appropriate use cases and contexts for implementation.

## The Future of Text Messaging in Health

So the simple text message works—as all those businesses who use it for appointment reminders will tell us and the wealth of published studies show us. But despite the well-established evidence for text messaging in health, there is always pressure to focus on newer technologies. While the true benefit of digital health lies in its future potential for more accessible and personalized health care, many of those in highest need may be unlikely to be able to access these newer technologies, such as wearables, at this time. These continued advancements risk excluding those with limited digital access, literacy, confidence, and motivation, thus exacerbating existing inequities in health outcomes [53,54]. To ensure all people are able to benefit from digital health, it is essential that equity remains at the forefront among digital health researchers, developers, and implementers. While the barriers to digital inclusion are addressed for these newer modalities, text messaging will continue to play a pivotal role in the delivery of equitable digital health tools to communities around the world. We believe that this is due not only to the technology’s low cost, vast reach, and low digital literacy requirements, but also its simplicity, which continues to appeal to end users among both consumers of health care and health professionals.

There is significant potential to update the simple text message—not by replacing it but by adding to the power of it. Examples are becoming more frequent, such as multimodal interventions incorporating newer technologies, such as wearables with text messaging [55,56], the development of algorithms to further enhance and personalize traditional text messaging programs [57,58], and algorithm and artificial intelligence–based tools using text messaging to alert or prompt health professionals or patients [59,60]. For example, a member of our team is developing a large language model text messaging program for smoking cessation powered by ChatGPT (OpenAI Inc). Such a program will provide users with both automated text messages and text messages from a digital chatbot coach. These types of interventions show promise in their ability to increase the complexity and sophistication of the content on the back end while maintaining the simplicity and accessibility of text messages for end users. Targeted research assessing the effectiveness of multimodal interventions is needed to understand whether and how these are more effective than interventions relying on text messages alone [61,62].

## Conclusion

In conclusion, we recommend that digital health researchers and developers do not overlook this simple yet effective modality in their search for the ultimate technological weapon in health. We challenge researchers to harness the learnings from this unassuming modality in the pursuit of equitable, accessible, and effective digital health solutions.

## Abbreviations

LSHTM: London School of Hygiene & Tropical Medicine
RCT: randomized controlled trial
STOMP: Stop Smoking by Mobile Phone
TextMATCH: Text for MATernal and Child Health
WHO: World Health Organization
